# Gaussian Process Regression for Predictive But Interpretable Machine Learning Models: An Example of Predicting Mental Workload across Tasks

**DOI:** 10.3389/fnhum.2016.00647

**Published:** 2017-01-11

**Authors:** Matthew S. Caywood, Daniel M. Roberts, Jeffrey B. Colombe, Hal S. Greenwald, Monica Z. Weiland

**Affiliations:** ^1^The MITRE Corporation, McLeanVA, USA; ^2^Department of Psychology, George Mason University, FairfaxVA, USA

**Keywords:** EEG, BCI, Gaussian Process Regression, machine learning, neuroergonomics

## Abstract

There is increasing interest in real-time brain-computer interfaces (BCIs) for the passive monitoring of human cognitive state, including cognitive workload. Too often, however, effective BCIs based on machine learning techniques may function as “black boxes” that are difficult to analyze or interpret. In an effort toward more interpretable BCIs, we studied a family of N-back working memory tasks using a machine learning model, Gaussian Process Regression (GPR), which was both powerful and amenable to analysis. Participants performed the N-back task with three stimulus variants, auditory-verbal, visual-spatial, and visual-numeric, each at three working memory loads. GPR models were trained and tested on EEG data from all three task variants combined, in an effort to identify a model that could be predictive of mental workload demand regardless of stimulus modality. To provide a comparison for GPR performance, a model was additionally trained using multiple linear regression (MLR). The GPR model was effective when trained on individual participant EEG data, resulting in an average standardized mean squared error (sMSE) between true and predicted N-back levels of 0.44. In comparison, the MLR model using the same data resulted in an average sMSE of 0.55. We additionally demonstrate how GPR can be used to identify which EEG features are relevant for prediction of cognitive workload in an individual participant. A fraction of EEG features accounted for the majority of the model’s predictive power; using only the top 25% of features performed nearly as well as using 100% of features. Subsets of features identified by linear models (ANOVA) were not as efficient as subsets identified by GPR. This raises the possibility of BCIs that require fewer model features while capturing all of the information needed to achieve high predictive accuracy.

## Introduction

Neuroimaging methods, particularly inexpensive and non-invasive techniques such as electroencephalography (EEG) and functional near infrared spectroscopy (fNIRS), are increasingly being used to continuously assess the cognitive state of individuals during task performance, an example of Neuroergonomics (Parasuraman, 2003; Parasuraman and Rizzo, 2006). This information can be used to better understand the demands of the task being performed, assess the limitations of the individual, or be fed back into the system to adjust the task relative to the individual’s current state. The use of physiological data to assess operator state has also recently been described as a ‘passive’ brain-computer interface (BCI) (Zander et al., 2009; Zander and Kothe, 2011), in contrast to traditional ‘active’ BCIs which utilize physiological data to allow an individual to act on the outside world (Wolpaw and Wolpaw, 2012).

Workload, the demand on the individual’s attention and working memory, is a cognitive state of special interest for passive measurement during task performance. Cognitive Load Theory (CLT) (Sweller et al., 1998) for example, suggests that maintaining an optimal level of workload for a given task can assist in learning new material. Further, Coyne et al. (2009) incorporate CLT with Multiple Resource Theory (MRT) (Wickens, 2008), which distinguishes between different modes of mental demand, suggesting that real-time measurement of participant workload could be utilized to optimally redirect mental demand across the several modes of resources available, for example presentation of information in a spatial versus verbal code as delineated by MRT (see Coyne et al., 2009). Physiological measures of workload have been sought in a variety of tasks including N-back (Grimes et al., 2008; Baldwin and Penaranda, 2011; Ayaz et al., 2012; Brouwer et al., 2012), the Sternberg Memory Scanning Task (Wilson and Fisher, 1995; Baldwin and Penaranda, 2011), memory span tests (Baldwin and Penaranda, 2011; Chaouachi et al., 2011), the MAT-B multi-tasking scenario (Wilson and Russell, 2003b; Kothe and Makeig, 2011); and operational simulations (Wilson and Russell, 2003a; Ayaz et al., 2012). The extent of this literature reflects scientific awareness of the limitations of behavioral or subjective workload assessment techniques, including limited sensitivity (Gevins and Smith, 2003; Just et al., 2003), subjective bias, and intrusiveness.

EEG based workload monitoring has been explored using a variety of different machine learning approaches, including step-wise linear discriminant analysis (SWDA) (Wilson and Fisher, 1995; Wilson and Russell, 2003a), artificial neural networks (ANN) (Wilson and Russell, 2003a; Baldwin and Penaranda, 2011), naïve Bayes models (Grimes et al., 2008), and least-angle regression (Kothe and Makeig, 2011).

To estimate workload as defined above based on EEG spectra, we applied a supervised machine learning approach, performing a statistical regression to take processed neurophysiological signals as inputs and to predict the load parameter N from the N-back task as an output. Many previous projects seeking to predict mental workload have used a classifier rather than a regressor approach. For example Baldwin and Penaranda (2011) used three working memory tasks, each with two levels of imposed difficulty, Wilson and Fisher (1995) used a battery of tasks, each with two levels of difficulty, while both Wilson and Russell (2003b) and Kothe and Makeig (2011) used the Multi-Attribute Task Battery with two levels of difficulty. Wilson and Russell (2003a) distinguished between up to seven conditions, using three different simulated ATC tasks with three, three, and one level of difficulty, respectively. However, the condition levels were treated as categorical, with the authors using ANN and stepwise discriminant analysis as classifiers to discriminate between data from each condition. Similarly, Grimes et al. (2008) predicted working memory load within 4 levels of the N-back task (0- through 3-back), but classified the levels as categorical labels, rather than as a continuous construct.

Treating mental workload as a series of categorical states has the effect of forcing estimates of workload to reside in discrete categorical bins without any continuous variation. The N-back task is comprised of discrete task load levels *N* = 1, 2, 3, and considered in isolation, this task is readily amenable to prediction based on a classifier. However, we conceptualize the mental state of workload as potentially lying along a continuum of values that the N-back task visits at discrete levels due solely to the structure of the task, not necessarily due to the inherent structure of working memory and attentional resources. The neurophysiological data is continuous in nature, and in order to preserve any potential information about workload as a continuously varying mental state, we treated the predicted N as a continuous variable even though all the training data for N was discrete. This required the use of a regression method rather than a classification method. One consequence of treating workload as a continuous measure is that the appropriate measure of error to be minimized in supervised training, as well as for operational testing, is continuous rather than discrete. For this reason, we present predictor performance primarily in standardized mean square error (sMSE), discussed more fully in the Section “Materials and Methods.”

Our choice of regression on a continuous task load variable was also motivated by a follow-on application of methods described here for estimating cognitive workload in a highly realistic en-route air traffic control (ATC) simulation, in which task difficulty was multivariate, and in each dimension highly granular and ordinal. This required a regressor rather than a classifier. The results presented here are meant to relate workload estimation to the dominant baseline literature on workload, and to generalize those studies to a broad variety of operational contexts including but not limited to ATC.

We employed Gaussian Process Regression (GPR; Rasmussen and Williams, 2005), a type of non-parametric regression, in which a single unknown target variable’s status (in this case, the number ‘N’ back) is estimated as a function of the state of one or more known input variables (in this case, power spectra at each electrode in the EEG montage).

Parametric regression methods, for example multiple linear regression (MLR), replace training data with a user-specified function, such as a line or curve or surface in the geometric space of inputs and outputs, whose parameters can be fitted to optimize estimation of outputs from inputs over the training data. For parametric methods, after the regression weights have been obtained, the original training data may be discarded. Non-parametric regression methods, by contrast, may keep the original training data to use as a scaffold for constructing a regressor function. Test data is compared to the training data points, with output value of the test point estimated via the distance of the test data input to the training data input. As a result of this weighting, estimates of output values form a locally smooth surface spanning the input data, in a process often referred to simply as smoothing. Non-parametric regression only assumes that data points with similar input values will be close in the output space. For GPR specifically, the form of the local weighting is defined by the covariance function and associated hyperparameters learned during model training.

This non-parametric GPR approach has several benefits with respect to cognitive monitoring. First, GPR makes few assumptions about the shape of the estimator function beyond the assumptions associated with the choice of covariance function. This is beneficial especially in high-dimensional input spaces, as is the case when there are many known variables for each data point, and the shape of the relationship between knowns and unknowns cannot easily be visualized and understood by a researcher.

Second, a GPR model can be constructed to change the width of the local weighting functions separately for each known input dimension during training, providing an indirect measure of that input dimension’s relevance. Measuring relevance adds interpretability to the model, and can be used to relate the features used by the model to existing literature, or aid in understanding which of the input variables could be left out of the analysis with little or no reduction in predictive accuracy.

A third benefit of GPR is its robustness to spontaneous failure of sources of input during operational test use of a BCI, such as the loss of good electrical contact by an EEG electrode or other equipment failure. Changes in the set of features available to machine learning methods challenge parametric methods such as linear or quadratic models, which typically have dependencies between features. In contrast, GPR depends more directly on the data and is robust to such changes; it can even be applied to data containing many fewer features than the model received during training.

Finally, a fourth major benefit of GPR for cognitive monitoring is its inherently probabilistic nature, returning both point predictions and confidence intervals around those predictions. Confidence values associated with each prediction may be used to dynamically inform decisions about when to trust a trained model’s predictions in operational settings.

While GPR has been used to classify EEG in the context of a BCI task involving imagined hand movement (Zhong et al., 2008; Wang et al., 2009), its use in cognitive state assessment has been limited (although see Chaouachi et al., 2011, 2015).

A reasonable assumption in cognitive neuroscience is that similar regions of the brain are engaged in similar functions across individuals during a specific task. This assumption motivates an approach to research that seeks constant neurophysiologic signatures for cognitive functions that generalize broadly among human participants. The present study has employed a more conservative and directed approach based on another reasonable assumption, which is that brain function involves learning, and that as a result, meaningful idiosyncratic differences may be expected among individuals with different learning histories, or within an individual over learning timescales. As such, we focused our analysis on a same-day, same-individual construct for training and testing our machine learning methods. Further, we did not set out to evaluate the neural basis of working memory and attention during task loading, although we regard this as an important goal for other research. Our goal was simply to evaluate the effectiveness and interpretability of a best-of-class machine learning approach for real-time, passive BCI targeted to cognitive monitoring in its simplest and most direct form.

We present a paradigm for assessment of cognitive workload for an operator, specifically the working memory and attentional demand based on measurable task load. We predict workload within several N-back tasks by training a GPR model, then testing it on held-out data from the same participant and session. The N-back task variants, which were designed to have face validity to an operational ATC task, include the following variations: auditory, numeric, and spatial. Finally, we analyze the GPR model to identify which EEG electrode sites, frequency bands, and derived features are essential to the predictive accuracy of the model, which serves to set a lower bound on the number of features required for accurate prediction.

## Materials and Methods

### Participants

The study included 16 male participants, aged 39–62 years old, selected for operational experience in the target operational domain of ATC. All participated voluntarily, and provided written informed consent after having had the procedures of the study described to them. Personally identifiable information for all participants was anonymized and kept secure by a trusted agent. All participants were salaried employees of the MITRE Corporation, and were compensated by allowing them to apply the time spent participating in the study to their work hours. Human subjects procedures were approved by the MITRE Corporation Institutional Review Board (MIRB), to which the Code of Federal Regulations, Title 45 (Public Welfare), Department of Health and Human Services, Part 46 (Protection of Human Subjects) applies for federally funded research involving human subjects.

### Task

To change working memory load in a controlled manner, we used an N-back working memory task in one of three stimulus modes (Auditory, Numeric, Spatial) and three task levels (*N* = 1, 2, 3) for each mode. The N-back task required participants to view a series of stimuli and press the spacebar key when the currently presented stimulus matched the stimulus presented N stimuli before the current one. The task was implemented in BrainWorkshop (Hoskinson, 2011), modified to synchronize with the EEG system.

**FIGURE 1:**
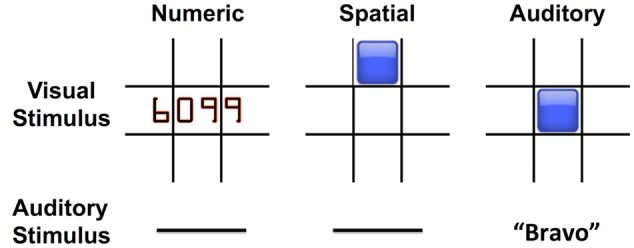
**N-back working memory tasks used three stimulus modes: Numeric, Spatial, and Auditory.** The visual and auditory stimuli associated with each mode are shown.

The Auditory stimuli were NATO letters (‘Alpha,’ ‘Bravo,’ ‘Charlie,’ etc.) spoken by a computer-generated voice. Numeric stimuli were numbers of 3 or 4 digits, e.g., “505” or “6099,” presented in the center of the screen. Spatial stimuli were blue squares presented in one of eight spatial locations on the screen, in a 3 × 3 grid leaving out the center square (**Figure 1**). Within each condition, eight unique stimuli were presented over the course of the block. Within the spatial condition, these eight stimuli were the aforementioned eight spatial positions. Within the Auditory and Numeric blocks, these eight stimuli were eight sounds or images randomly selected from a pool of 26 possible NATO letter sounds or 26 possible Numeric images. Each trial lasted 3 s, with visual stimuli in the Numeric and Spatial conditions remaining onscreen for the first 500 ms of the trial. The stimuli for each trial were selected pseudorandomly from the eight possible stimuli within the block, with an N-back match additionally forced on 1/8 of trials. The combination of the inherent 1/8 probability of random match and the independent forced match probability of 1/8 results in an overall 76.56% (7/8 ^∗^ 7/8) chance of non-matching stimuli and 23.44% chance of matching stimuli. Participants were instructed to respond to matching stimuli by pressing the spacebar key on a standard computer keyboard, while non-matching trials did not require a response.

The task was performed in blocks of 100 comparison trials of a single modality and task level. Participants were allowed to take short breaks between 100 trial blocks. Three 100-trial blocks of each N-back level were performed for each of three stimulus modes, totaling 900 trials for each participant. Stimulus modes were counterbalanced across participants, while N-back levels were performed in the order 1-Back, 2-Back, 3-Back, within each modality block. Before each block, a resting baseline condition was recorded, however, data from this resting baseline condition was not included in the regression models. Prior to the experimental blocks, participants completed 20 practice trials at each N-back level of a Color variant, in which participants indicated if the color of the current stimulus (a square presented in the center of the display) matched the color of the stimulus presented N stimuli prior.

Following each block, participants reported their subjective rating of block difficulty (subjective workload) on a 1–7 Likert scale from low to high workload. Subjective workload ratings were collected in order to confirm that the N-back task was subjectively experienced as more demanding as N-back level increased, as well as to investigate any subjective differences in demand between N-back modalities used (Auditory, Numeric, Spatial).

#### Behavioral Data

Accuracy on the N-back task is evaluated within each block as the number of true positive (TP) responses (correctly responding when the current stimulus matched the stimulus presented “N” back), divided by the sum of the TP responses, false positive (FP) responses (incorrectly responding when the current stimulus did not match the stimulus presented “N” back), and false negative (FN) responses (incorrectly failing to respond when the current stimulus matched the stimulus presented “N” back). This is equivalently described as accuracy = TP/(TP + FP + FN). As the N-back match probability was 23.44%, this places an upper limit of chance performance at 23.44%. For example, responding to all stimuli regardless of N-back match would generate an accuracy of 23.44%, while responding to no stimuli regardless of N-back match would generate an accuracy of 0%.

Behavioral accuracy and subjective workload were assessed via separate two-way repeated-measures ANOVAs, with factors N-back level (1, 2, 3) and task mode (Auditory, Numeric, Spatial). Mauchly’s test was used to assess sphericity, with *F*-values adjusted via Greenhouse–Geisser correction where appropriate. Effect size is indicated by generalized eta squared ($\eta_{G}^{2}$) (Olejnik and Algina, 2003), a measure of effect size appropriate for repeated measures designs (Bakeman, 2005).

### EEG Collection

EEG data were collected via a 32-channel actiCAP active electrode system and BrainAmp amplifier at a sampling rate of 500 Hz using Recorder software (Brain Products GmbH), with online reference at electrode FCz and online bandpass filter from 0.1 to 250 Hz.

#### Processing

Oﬄine data analysis was completed with the EEGLAB toolbox for MATLAB (Delorme and Makeig, 2004) and custom MATLAB scripts. EEG signals were band-pass filtered to 1–50 Hz, down-sampled to 250 Hz, and re-referenced to the average of the left and right mastoid sites (TP9 and TP10).

Trial epochs were extracted from 0 to 3 s post stimulus onset, and labeled according to N-back level, stimulus mode, and behavioral accuracy. Channels and epochs containing paroxysmal artifacts such as gross EMG or cap movement were identified via visual inspection, and were removed from further analysis (Delorme et al., 2007). Between 0 and 4 electrodes were removed per participant (mean of 0.75 electrodes were removed). The remaining epochs were decomposed via independent component analysis (ICA), using the extended InfoMax algorithm as implemented in EEGLAB. For each participant, independent components (IC) representing sources of artifact including eye blinks, lateral eye movements, and muscle activity were manually identified based on IC topography, frequency spectra, and time-domain activity, and were removed from the data.

#### Feature Extraction

Band-power features were extracted by transforming each epoch from the time to frequency domain via the Welch method. The Welch method averages the Fast Fourier Transform (FFT) results from several overlapping Hamming windowed segments. A window size of 500 points (2 s) and overlap of 250 points (1 s) were used, along with a 512 point FFT.

For each channel, frequencies were averaged into 6 pre-specified bands, delta: 1–3 Hz, theta: 4–7 Hz, low alpha: 8–10 Hz, high alpha: 11–12 Hz, beta: 13–25 Hz, gamma: 26–40 Hz.

Band power values were then converted to the natural logarithm of their original values to more closely approximate a Gaussian distribution, and each feature was then zero-centered and normalized by its standard deviation on the training set. The same normalization was applied to trials from both training and test sets; both were *z*-scored relative to the mean and standard deviation of the training set. Trials from the test set were *z*-scored relative to the mean and standard deviation of the training set, rather than the test set, to place the test trials on the same scale as the training set. Scaling the test set trials to the test set mean and standard deviation could eliminate meaningful differences that could be present between training and test sets. For example, when using a trained model to derive workload predictions on a new task that is on average more difficult than the training task. In addition, for online prediction applications the mean and standard deviation of the full test set are unknown in advance.

### Machine Learning: Gaussian Process Regression

The Information present in EEG band-power features about task level was analyzed using a continuum of methods including ANOVA, MLR and GPR. Additionally, while imposed task level (the number ‘N’ back) was of primary interest, models were additionally constructed using participants’ subjective rating of their mental demand as labels.

For machine learning, a feature vector composed of each of the 6 bands at each of the 32 electrode sites, less any electrodes rejected due to excessive artifact or poor electrode contact, was taken as input. The features were normalized as described in Section “Feature Extraction.” The length of the feature vector is the product of the number of bands and number of electrode sites analyzed, and was thus of length 192 (6 ^∗^ 32) for the 11 participants for whom no electrodes were rejected due to artifact, and six elements (frequency bands) less for each rejected channel for the remaining five participants.

#### Gaussian Process Regression

A GPR model, a form of Bayesian non-linear regression, was trained using the Gaussian Processes for Machine Learning (GPML) library for MATLAB (Rasmussen and Williams, 2005; Rasmussen and Nickisch, 2010). A GPR model is defined primarily by the selection of a covariance function, which defines how the expected value of the target variable changes as values change across the input space. Here, a squared-exponential covariance function with automatic relevance determination (ARD) was used, in conjunction with a constant zero mean function. ARD refers to the inclusion of a length-scale for each feature within the covariance function, which can be examined after training to determine the relative importance of that feature to prediction. As described by Rasmussen and Williams (2005), the squared exponential covariance function with ARD is defined as:

(1) $$\begin{array}{c} k(x_{p},x_{q})=\;\sigma_{f}^{2}*\exp\left(-\frac{1}{2}{\left(x_{p}-x_{q}\right)}^{⊺}*(diag{\left(\ell \right)}^{-2})*(x_{p}-x_{q})\right) \end{array}$$

Where x_p_ and x_q_ represent values in the input space, $\sigma_{f}^{2}$ represents the noise free signal variance, and ℓ is a vector of length-scales (one for each feature).

This covariance function is stationary in the sense that the relationship between values in the input space depends only on their distance, not to their particular location in the space. The squared exponential covariance function was selected *a priori* based on its relative simplicity, the assumption inherent in its use is that data points that are close in the input space will tend to be close in the output space. The constant zero mean function was selected as the data was normalized to have zero mean in the training set. Rasmussen and Williams (2005) present an in-depth presentation of the properties of different covariance and mean functions in the context of GPR.

The covariance and mean functions were used in conjunction with a Gaussian likelihood for prediction via the following equations, all from Rasmussen and Williams (2005):

(2) $$\begin{array}{c} f_{*}|X,y,X_{*}\sim N({\bar{f}}_{*},cov(f_{*})) \end{array}$$

Where f_∗_ is a posterior distribution, *X* is a matrix of training inputs, *y* is a vector of training targets, X_∗_ is a matrix of test inputs, 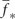 is the posterior mean, and cov(f_∗_) is the posterior covariance.

The posterior mean is specified as:

(3) f*¯=Δ EEE[f*|X,Y,X*]=K(X*,X)[K(X,X)+σn2I]−1y

The posterior covariance is specified as:

(4) $$\begin{array}{c} cov(f_{*})=\;K(X_{*},X_{*})-K(X_{*},X){\left[K(X,X)+\sigma_{n}^{2}I\right]}^{-1}K(X,X_{*}) \end{array}$$

Where *K* indicates a covariance matrix, and $\sigma_{n}^{2}$ is a noise variance term.

The covariance function contains several hyperparameters, which are optimized during model training. Hyperparameters for the covariance function include a length-scale for each feature (ℓ), and a noise free signal variance ($\sigma_{f}^{2}$). In addition, the covariance function is evaluated using a Gaussian likelihood function, which has a single hyperparameter, the noise variance ($\sigma_{n}^{2}$). The constant zero mean function has no hyperparameters.

Prior to each model run, these hyperparameters are set to default values, which are subsequently adjusting during model training. Here, for hyperparameters associated with the squared exponential covariance function with ARD, the length-scale for each feature was set to 10, and the signal variance was set to 1. Additionally, for the hyperparameter associated with the Gaussian likelihood function, the likelihood variance was set to 1. These hyperparameters are then optimized within each model run by the GPML library, by minimizing the negative log marginal likelihood on the training set, over 100 function evaluations.

After training the model, new predictions are made via the conditional distribution of target output values, given the test inputs, training inputs, training targets, covariance function, and associated hyperparameters. The mean and variance of the posterior target distribution are used to generate point predictions and confidence intervals, respectively.

#### Evaluation of Model Performance

Model performance at predicting N-back task level (N) was assessed via fivefold cross-validation with a five trial buffer between training and test sets. Data from each modality and N-back level block (9 blocks total) was split into five partitions, with each partition containing a contiguous block of trials. On any given fold of the fivefold cross-validation procedure, 4 of the 5 partitions (80% of data) were used for training the GPR model, with the remaining partition held out as a test set for assessing model performance. Additionally, any trials from the test set that occurred within five trials of a member of the training set were removed from the test set and not included in measures of model performance. Trials were removed from the test set, and not the training set, to ensure a constant amount of training data (4 of 5 partitions or 80%) across runs. These neighboring trials were removed in order to reduce any short-time scale effects of attention or participant posture on model performance. After identification of the training and test trials from each of the 9 blocks, the data, from these 9 blocks (3 N-back levels and 3 modalities) were pooled for training and testing, labeled by N-back level and subjective workload rating provided by each participant after each block, but not labeled by modality. Data from the three modalities were pooled in an attempt to identify features indicative of working memory load independent of any particular stimulus modality. Measures of prediction quality were obtained for each participant by combining the results from the five model runs. Specifically, for each participant, the true and predicted values from each model run of collected and used to compute a single sMSE and a single Pearson correlation coefficient for that participant. On average, 661.25 trials were included in each training set, and 88.66 trials were included in each test set. Despite the use of fivefold cross-validation, the number of trials in the average test set is less than 1/4 of the trials in the average training set due to the removal of trials from the set test partitions that occurred within five trials of a trial from the training set.

As a parametric regression model for performance comparison to GPR, we used MLR with one linear term per feature plus a constant term. The model training and testing functions were implemented using BCILAB (Kothe and Makeig, 2013). Our BCILAB plugins for Gaussian Processes (a BCILAB wrapper around the GPML library), and for MLR (a BCILAB wrapper around the ‘regress’ function in MATLAB), are available as open source code.

Continuous prediction accuracy was quantified using two metrics: standardized mean squared error (sMSE) and Pearson correlation coefficient (*r)*. sMSE is the mean squared error (MSE) of true and predicted values, divided by the variance of the true values. sMSE has a characteristic scale of 0–1 and, due to the standardization on the variance of the true values, is dimensionless, unlike the MSE. Like MSE, sMSE equals 0 for a perfectly accurate prediction. However, due to standardization sMSE equals 1 for a naïve model which always predicts the mean of the ground truth values, and exceeds 1 for predictions that are more erroneous than could be obtained by only predicting the mean of the ground truth values. For machine learning purposes, *r* ranges from 1 (perfect accuracy) to 0 (uncorrelated); however, a naïve model predicting a constant output will show positive *r*. Additionally, although mental workload is argued to be best treated as a continuous, rather than discrete, variable, we have also included discretized versions of the continuous MLR and GPR output. These predictions were included to allow the presented results to be more readily compared with other reports in which discrete classification is performed, and are computed by rounding each continuous prediction to the nearest label in the training set (i.e., a continuous prediction of 2.4 is relabeled as 2), then computing the fraction of predictions which have the correct label.

While predicting the imposed task load is of primary focus, an additional model was trained to predict subjectively experienced workload, using the reports provided by each participant following each task block. This model was computed in the same manner as the previously described model for imposed task load, with the exception of each trial being labeled according to the subjective workload provided by that participant for that block (a value that can range from 1 to 7), rather than the imposed task load (1–3).

Additionally, models were constructed with data from single task variants, in order to investigate the ability of the model to predict the task load within task variants relative to across task variants. Data from each task variant and load was split into five partitions, with a separation of at least five trials between partitions, as previously described for the primary analysis. While the primary analysis combined data across the three task variants for a given fold, the present analysis used data from only a single task variant for training, and a single task variant for testing. For example, the first run of training on the Auditory task and testing on the Auditory task uses the first training fold and first testing fold of exclusively Auditory task data. In contrast, the first run of training on Auditory task and testing on Spatial task uses the first training fold of exclusively Auditory task data, and first testing fold of exclusively Spatial task data. As three task variants were included in the experiment, generating nine combinations of training and test task variants.

### Feature Analysis

To illuminate the association between individual participants’ EEG features and working memory load prediction, we used two techniques. First, we applied a one-way ANOVA for task level (the number N-back; 1, 2, 3) to individual participants’ EEG data. Second, using the trained GPR predictive model, we examined ARD length scales of each feature to identify which played the greatest role in prediction. ARD length scales were also used to evaluate the predictive power of alternate EEG electrode montages mapped to other commercial EEG equipment, as is further explained in Section “Results.”

## Results

### Behavioral Data

**FIGURE 2:**
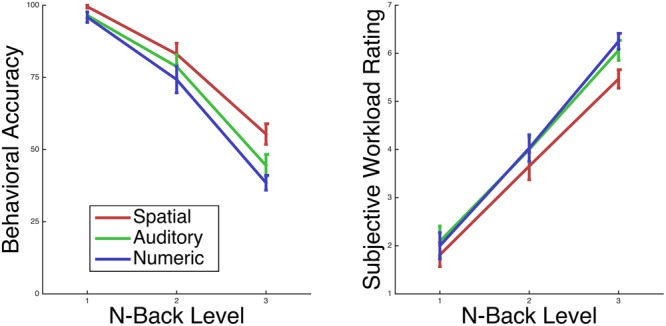
**Participant performance and subjective workload on the N-back task changed monotonically with level**.

Participants completed all N-back working memory tasks (Auditory, Numeric and Spatial tasks) at above chance performance within all N-back levels. As N-back level increased, performance significantly decreased. Across all participants and modalities, mean 1-back performance was 97%, mean 2-back performance was 79%, and mean 3-back performance was 46% (**Figure 2**). There was a main effect of level, *F*(2,30) = 135.108, *p* < 0.001, $\eta_{G}^{2}$ = 0.747, as well as a main effect of mode, *F*(2,30) = 14.457, *p* < 0.001, $\eta_{G}^{2}$ = 0.097, and a level by mode interaction, *F*(4,60) = 3.336, *p* = 0.016, $\eta_{G}^{2}$ = 0.033.

As indicated by the reported measure of effect size generalized eta squared ($\eta_{G}^{2}$), the effect of N-back level on performance was of greater magnitude than the effect of modality of performance.

Participants reported subjective workload levels spanning from 1 to 7, with mean 1-back workload 2.0, mean 2-back workload 3.9, and mean 3-back workload 5.9 (**Figure 2**). For subjective workload, there is both a main effect of level, *F*(2,30) = 181.449, *p* < 0.001, $\eta_{G}^{2}$ = 0.735, and a main effect of mode, *F*(2,30) = 7.773, *p* = 0.002, $\eta_{G}^{2}$ = 0.042, while the level by mode interaction was not significant (*p* > 0.10).

Similar to task accuracy, according to the reported measure of effect size, generalized eta squared, the effect of N-back level on subjective workload was of greater magnitude than the effect of modality on subjective workload. Participants performed the Spatial task more accurately, and additionally rated it as lower in subjective workload, in comparison to the Auditory or Numeric tasks. Although each task used only 8 stimuli within each block, it is possible that the consistent use of the same 8 spatial locations across blocks of the spatial task contributed to this performance and subjective workload difference.

### Predictive Accuracy of BCI

The GPR with ARD was trained to predict task level for individual participants on a mixture of all three N-back tasks, and tested on the left-out test data using fivefold cross-validation.

**FIGURE 3:**
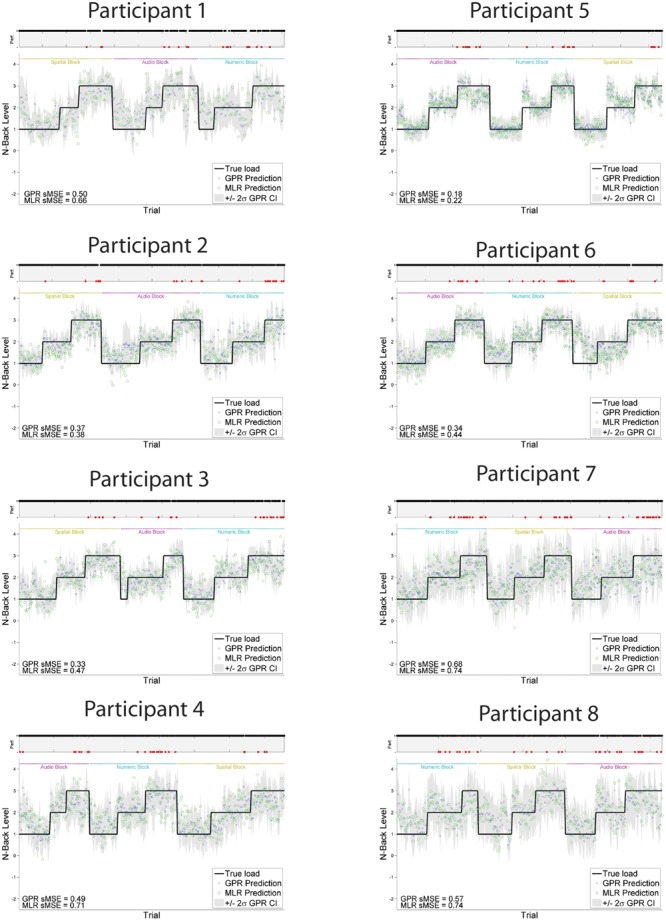
**GPR predictions of N-back level for participants 1–8**. The figure displays predictions derived from the 5 cross-validation folds in a single graph. Main graph shows ground truth task load (black line). Predicted load is represented as the point prediction for each trial, for both Gaussian Process (GP) and Multiple Linear Regression (MLR) predictors, displayed in blue x’s and green o’s, respectively. Task block for each section of the experiment (Auditory, Numeric, Spatial) is indicated by colored labels above the predictions. The gray region shows the ± 2σ confidence interval for each GP point prediction generated by the model. sMSE for both GP and MLR models are included in the lower left of each participant panel. Participant behavioral performance is shown in the line graph at top of each subplot (+ = correct, - = incorrect, with incorrect points also colored in red) in order to visually examine the relation between model prediction and participant behavioral performance on the task.

**FIGURE 4:**
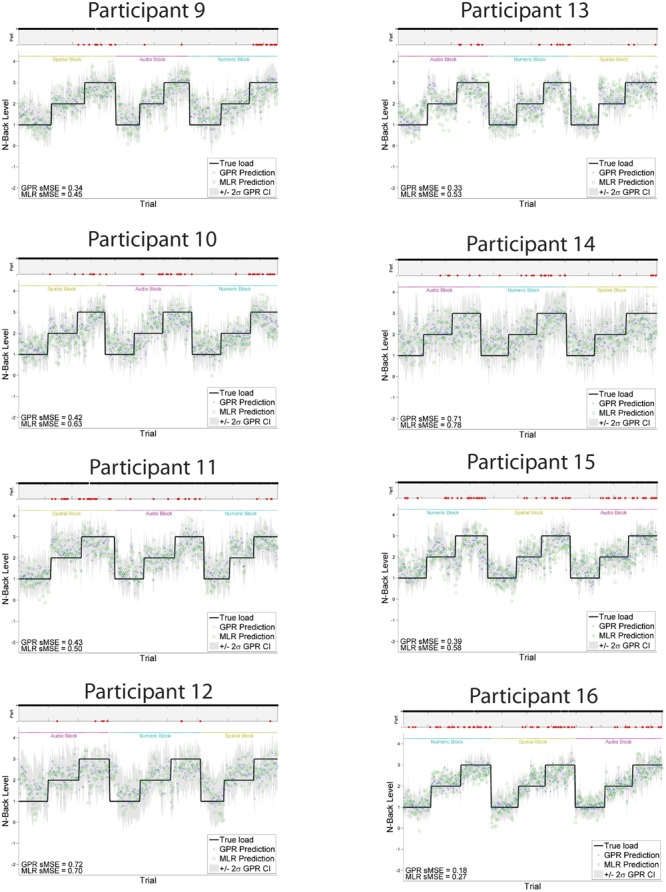
**GPR predictions of N-back level for participants 9–16.** The figure displays predictions derived from the 5 cross-validation folds in a single graph. Main graph shows ground truth task load (black line). Predicted load is represented as the point prediction for each trial, for both Gaussian Process (GP) and Multiple Linear Regression (MLR) predictors, displayed in blue x’s and green o’s, respectively. Task block for each section of the experiment (Auditory, Numeric, Spatial) is indicated by colored labels above the predictions. The gray region shows the ± 2σ confidence interval for each GP point prediction generated by the model. sMSE for both GP and MLR models are included in the lower left of each participant panel. Participant behavioral performance is shown in the line graph at top of each subplot (+ = correct, - = incorrect, with incorrect points also colored in red) in order to visually examine the relation between model prediction and participant behavioral performance on the task.

Individually trained GPR models were able to predict task level across participants with high accuracy. sMSE mean and standard error across multiple participants was 0.44 ± 0.04, where 0 is perfect prediction and 1 is a model which performs no better than a naive model always predicting the mean of ground truth (**Table 1**). Pearson’s *r* correlation was 0.75 ± 0.03, where *r* = 1 is perfect, *r* = 0 is uncorrelated. (All error estimates are given as standard error of the mean.) The GPR predictions of task level for each trial are presented within **Figure 3** (participants 1–8) and **Figure 4** (participants 9–16). The predictions derived from the 5 model folds have been merged into a single dataset for presentation.

**Table 1:** Predictive ability of feature subsets.

	max # features	Correlation *r*	Standardized MSE
**Feature subset**			
All	192	0.75 ± 0.03	0.44 ± 0.04
**GPR-ARD subsets**			
50% shortest length scales	96	0.75 ± 0.03	0.44 ± 0.04
25% shortest length scales	48	0.74 ± 0.03	0.46 ± 0.04
**ANOVA subsets**			
Top 50% ANOVA features	96	0.73 ± 0.03	0.47 ± 0.05
Top 25% ANOVA features	48	0.68 ± 0.03	0.59 ± 0.06
**Electrode site subsets**			
B-Alert X-10 channels	54	0.53 ± 0.04	0.72 ± 0.04
Emotiv EPOC^a^ channels	96	0.73 ± 0.03	0.46 ± 0.04
Parietal channels only	54	0.56 ± 0.04	0.68 ± 0.05
Occipital channels only	30	0.52 ± 0.04	0.72 ± 0.05
**Frequency band subsets**			
Delta	32	0.21 ± 0.03	0.95 ± 0.02
Theta	32	0.23 ± 0.04	0.94 ± 0.02
Low alpha	32	0.24 ± 0.04	0.92 ± 0.03
High alpha	32	0.25 ± 0.04	0.93 ± 0.02
Beta	32	0.65 ± 0.03	0.57 ± 0.05
Gamma	32	0.74 ± 0.03	0.45 ± 0.04

*Feature subsets are categorized by whether they are subsets of electrode sites (over all frequency bands), frequency bands (at all electrode sites), or selected from sites × bands. ^a^For this analysis, Emotiv EPOC sites AF3 and AF4, which were not included in our configuration, were substituted by adjacent sites Fp1 and Fp2.*

**FIGURE 5:**
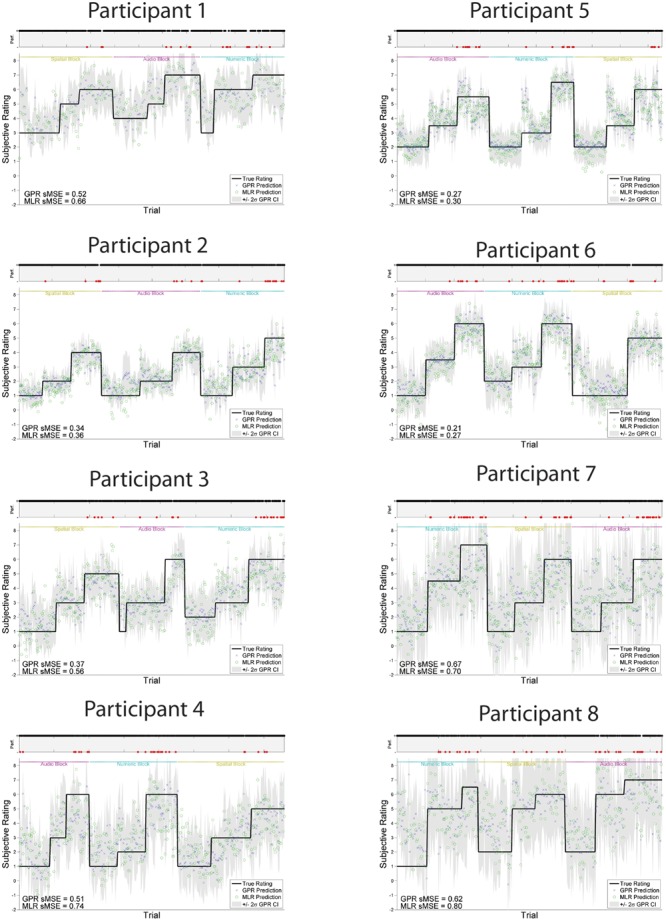
**GPR predictions of subjective workload for participants 1–8.** The figure displays predictions derived from the 5 cross-validation folds in a single graph. Main graph shows ground truth subjective workload (black line) provided by the participant at the end of the block. Predicted subjective workload is represented as the point prediction for each trial, for both Gaussian Process (GP) and Multiple Linear Regression (MLR) predictors, displayed in blue x’s and green o’s, respectively. Task block for each section of the experiment (Auditory, Numeric, Spatial) is indicated by colored labels above the predictions. The gray region shows the ± 2σ confidence interval for each GP point prediction generated by the model. sMSE for both GP and MLR models are included in the lower left of each participant panel. Participant behavioral performance is shown in the line graph at top of each subplot (+ = correct, - = incorrect, with incorrect points also colored in red) in order to visually examine the relation between model prediction and participant behavioral performance on the task.

**FIGURE 6:**
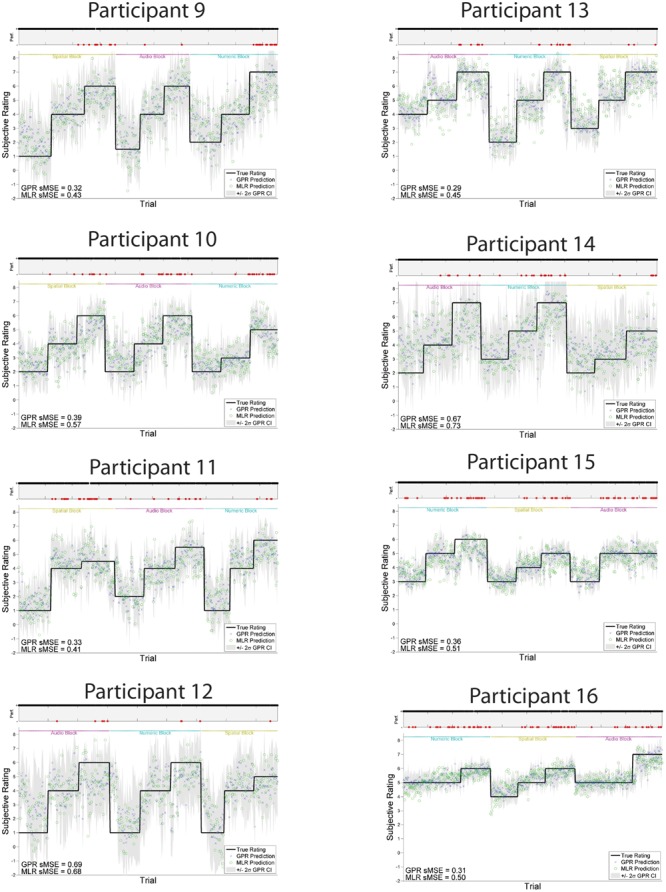
**GPR predictions of subjective workload for participants 9–16.** The figure displays predictions derived from the 5 cross-validation folds in a single graph. Main graph shows ground truth subjective workload (black line) provided by the participant at the end of the block. Predicted subjective workload is represented as the point prediction for each trial, for both Gaussian Process (GP) and Multiple Linear Regression (MLR) predictors, displayed in blue x’s and green o’s, respectively. Task block for each section of the experiment (Auditory, Numeric, Spatial) is indicated by colored labels above the predictions. The gray region shows the ± 2σ confidence interval for each GP point prediction generated by the model. sMSE for both GP and MLR models are included in the lower left of each participant panel. Participant behavioral performance is shown in the line graph at top of each subplot (+ = correct, - = incorrect, with incorrect points also colored in red) in order to visually examine the relation between model prediction and participant behavioral performance on the task.

The models trained using GPR and all features performed significantly better than models trained using MLR and all features, using the same training and test folds. GPR models had mean sMSE of 0.44 ± 0.04, while MLR models had mean sMSE of 0.55 ± 0.04, *t*(1,15) = -6.28, *p* < 0.001. Similarly, models trained to predict subjective workload ratings performed better using GPR than MLR. The subjective workload model trained using GPR had mean sMSE of 0.43 ± 0.04, while the analogous model trained using MLR had mean sMSE of 0.54 ± 0.04, *t*(1,15) = -6.07, *p* < 0.001. Measures of model quality in terms of Pearson *r* and discretized classification are provided in **Table 2** for comparison with other paradigms. The GPR predictions of subjective workload for each trial are presented within **Figure 5** (participants 1–8) and **Figure 6** (participants 9–16). The predictions derived from the 5 model folds have been merged into a single dataset for presentation. **Table 3** additionally displays the sMSE for each participant, both collected across the 5 runs prior to calculating sMSE, and the mean and standard deviation of sMSE calculated by first computing sMSE within run. The sMSE for each participant collected across the 5 runs prior to calculating sMSE is very similar to the mean of sMSE calculated by first computing sMSE within runs.

**Table 2:** Predictive ability of Gaussian Process Regression (GPR) model in comparison to multiple linear regression (MLR) model, predicting either task load or subjective workload using all model features.

Predicted Variable	Prediction method	*r*	sMSE	Classification
Task load	GP	0.75 ± 0.03	0.44 ± 0.04	0.70 ± 0.02
Task load	MLR	0.69 ± 0.03	0.55 ± 0.04	0.63 ± 0.02
Subjective workload	GP	0.76 ± 0.03	0.43 ± 0.04	0.52 ± 0.03
Subjective workload	MLR	0.70 ± 0.02	0.54 ± 0.04	0.44 ± 0.02

*Model performance is provided via Pearson’s correlation coefficient ‘*r’*, standardized mean squared error (sMSE), and categorical classification accuracy. Values displayed are the mean performance across participants ± the standard error of the mean across participants.*

**Table 3:** Individual participant GPR model performance.

Participant	Total sMSE	sMSE Mean Over 5 Runs	sMSE standard deviation over 5 runs
1	0.50	0.47	0.24
2	0.37	0.34	0.11
3	0.33	0.30	0.14
4	0.49	0.45	0.27
5	0.18	0.18	0.10
6	0.34	0.32	0.15
7	0.68	0.66	0.21
8	0.57	0.54	0.17
9	0.34	0.32	0.17
10	0.42	0.40	0.15
11	0.43	0.41	0.19
12	0.72	0.65	0.45
13	0.33	0.31	0.23
14	0.71	0.67	0.28
15	0.39	0.37	0.22
16	0.18	0.18	0.04

*The total sMSE combines the results of each run into vectors of true and predicted values, which weights each test example equally. The sMSE mean over 5 runs averages the sMSE results derived from each test run. The sMSE standard deviation is the standard deviation of the sMSE results derived from each test run. The total sMSE and sMSE mean are not equal because each test run can have different numbers of test trials, due to the removal of any test trials that occurred within 5 trials of a training trial.*

Comparing the performance of the GPR model trained on task level to the equivalent model trained on subjective workload, the ability to predict the two label types was not significantly different *t*(1,15) = 0.53, *p* = 0.606.

### Feature Analysis in the ANOVA, GPR, and MLR Models

To determine which EEG band-site features were significantly associated with N-back level, we applied a one-way ANOVA for level to individual participants’ EEG data. Because the predictive model was also individualized, it was necessary to analyze individual data rather than group effects as is commonly done in cognitive neuroscience.

Using the GPR predictive model, we examined the set of features to identify which played the greatest role in prediction. When ARD is used in training a GPR, the resulting length scale of each feature indicates the relative sensitivity of the model to changes in that feature’s value (MacKay, 2003; Rasmussen and Williams, 2005). A model is more sensitive to features with short length scales and least sensitive (most invariant) to features with long length scales.

**FIGURE 7:**
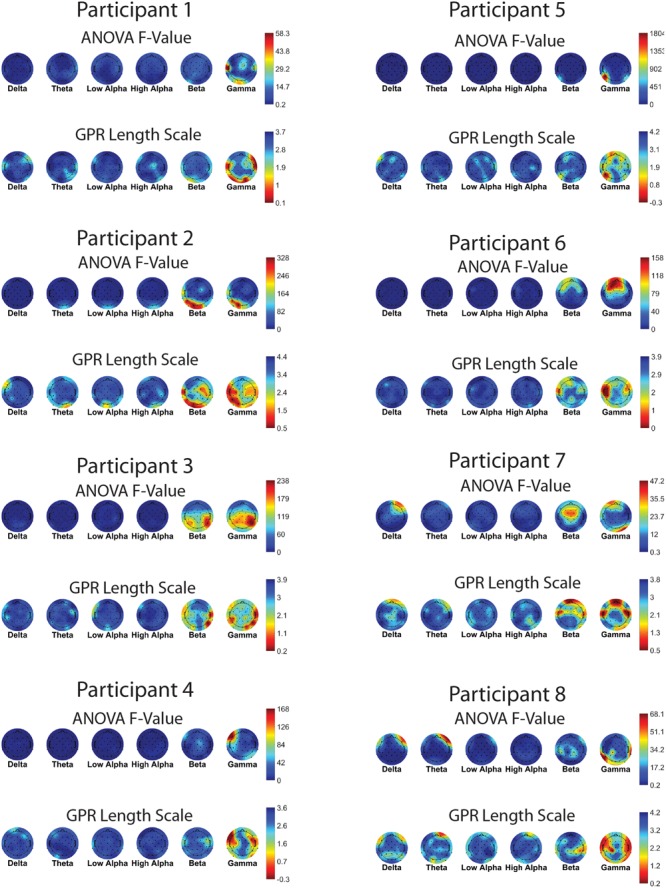
**EEG features (bands and sites) for participants 1–8.** Top of each participant plot: ANOVA *F*-values for memory load. Red (higher values) corresponds to larger *F*-value for feature. Bottom of each participant plot: log scaled Gaussian Process model length scales. Red (lower values) corresponds to higher sensitivity for feature. The directionality of the colormap is swapped between ANOVA and GPR in order for warmer (redder) colors to always indicate greater feature relevance.

**FIGURE 8:**
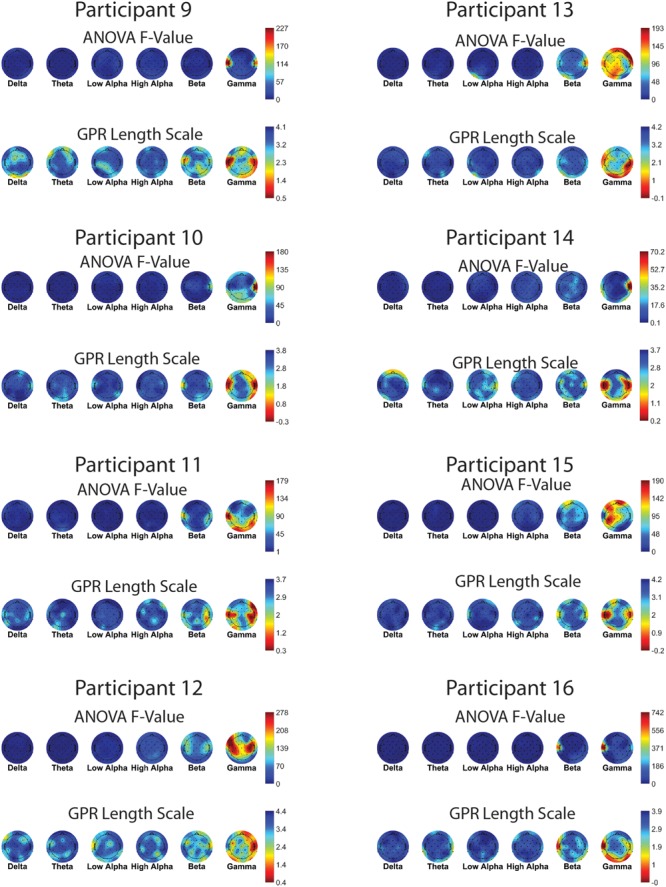
**EEG features (bands and sites) for participants 9–16.** Top of each participant plot: ANOVA F-values for memory load. Red (higher values) corresponds to larger *F*-value for feature. Bottom of each participant plot: log scaled Gaussian Process model length scales. Red (lower values) corresponds to higher sensitivity for feature. The directionality of the colormap is swapped between ANOVA and GPR in order for warmer (redder) colors to always indicate greater feature relevance.

**Figures 7** and **8** show one-way ANOVA *F*-values compared to GPR length scales for each channel × band power feature, for each of the 16 participants. The values displayed are the average of the values for that participant, over the 5 runs of cross-validation performed. Although there is substantial between-participant variability, gamma band features at occipital and temporal sites are commonly utilized by the GPR models for prediction.

Unlike the features with significant level effects in ANOVA, the (most sensitive) features with the shortest length scales are not generally clustered into individual frequency bands, with the exception of the gamma band, where several channels are uniformly short in length scale. This lack of spatial patterning was also typical across participants.

As the MLR predictions were derived from a multivariate regression, multicollinearity between features can make interpretation of the resulting regression coefficients difficult or misleading (Haufe et al., 2014). Weights from the MLR models were therefore transformed into activation patterns via Equation (6) from Haufe et al. (2014). Specifically, the activations are derived by:

(5) $$\begin{array}{c} A=\sum_{x}W\sum_{\hat{S}}^{-1} \end{array}$$

**FIGURE 9:**
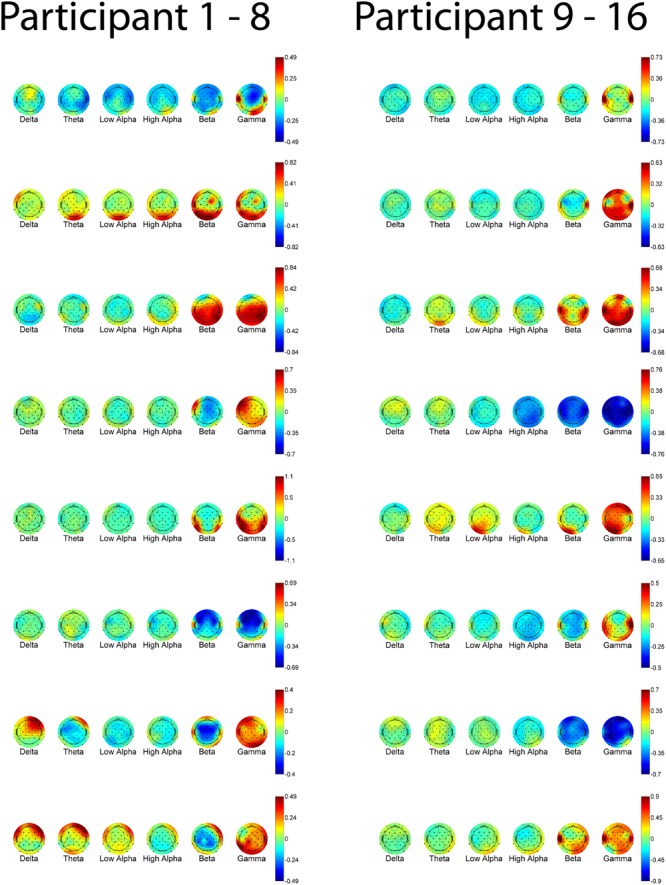
**Activations derived from the Multiple Linear Regression models predicting task load, at each band and electrode site for all participants.** Red values indicate more positive activations; blue values indicate more negative activations.

Where ∑ _x_ is the covariance of the data, *W* is the multivariate regression weights, and $\sum_{Ŝ}^{–1}$ is the inverse covariance matrix of the latent factors, in this case simply the N-back level labels. **Figure 9** displays the activation patterns from the MLR models predicting task load.

### Feature Selection and Prediction Accuracy

For each participant, we compared the predictive ability of several feature subsets. The feature subset “All” (i.e., all electrodes × all bands) was the upper bound on predictive accuracy for this data set (**Table 1**).

To illuminate which frequency bands are important to the task, we considered the predictive accuracy of feature subsets corresponding to single frequency bands (e.g., the 32 features corresponding to the beta band at all electrodes). While all bands contributed to predictive accuracy, the largest contribution came from features in the beta and gamma frequency range. This suggests that information that might be discounted by standard EEG analysis can be highly informative in the context of a BCI predicting workload.

How important was it for the BCI to include all 32 electrodes for this task? We considered feature subsets with a smaller number of EEG electrode sites than were actually measured (but all frequency bands). The model’s accuracy for several subsets of electrode sites, averaged over all 16 participants, is also shown (**Table 1**). We compared the montage of our laboratory EEG headset to the montage of two EEG headsets including fewer electrodes, one focused on rapid deployment (B-Alert X10) and one on affordability for home use by consumers (Emotiv EPOC). In this task, the 16 channels present in the Emotiv EPOC device, primarily near equatorial sites such as F7, F8, P3, P4, P7, and P8, capture much of the model’s predictive ability. However, the montage of channels present in the B-Alert X-10, more along midline sites such as Fz, Cz, and POz are less effective, generating similar performance as achieved by only looking at a single region’s channels (e.g., parietal channels or occipital channels).

One operationally relevant scenario is that a full laboratory electrode cap might be used to calibrate a model for a participant before switching to a simpler EEG device for operational use. We tested this concept by training the GPR model on the full feature set, leaving out features using ARD or ANOVA F-values, then testing the newly reduced model’s predictive power. With this paradigm, we found that selecting a reduced feature model using GPR length-scales was more resilient than reducing models using ANOVA features. For both the top 25% and top 50% of features, selection based on training data GPR length-scale generated a model with lower test set sMSE in comparison to selection based on training data ANOVA *F*-value, as evaluated with paired samples *t*-tests; [*t*(1,15) = -5.62, *p* < 0.001 for the top 25% of features, *t*(1,15) = -2.68, *p* = 0.017] for the top 50% of features, see **Table 1**.

**FIGURE 10:**
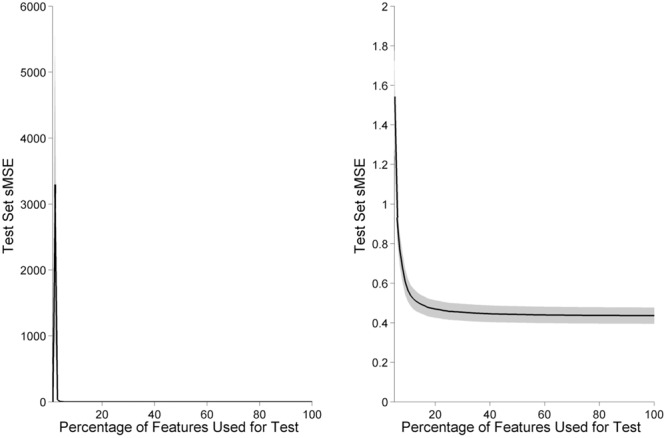
**Model accuracy increases as features are added in order of informativeness.** Black line indicates model performance as the percentage of features are increased, averaged across participants. Gray shading indicates the standard error of the mean, across participants. Below a minimum number of features, model error increases due to bias from insufficient dimensionality. After approximately the top 20% of features have been included in the model, adding additional features provides little improvement to model performance. The left panel displays 1–100% of features, while for clarity the right panel displays only 5–100% of features, where the GPR model is able to function.

A similar method allowed us to measure the absolute minimum number of features required for prediction of task level. Each individual’s feature length scales were sorted from shortest to longest, and the GPR model was tested on subsets of increasing size, from 1 to 100% of total features, in increments of 1% (**Figure 10**). As features are added beyond the minimum level required for the model to function, the trend is for classifier error to decrease monotonically until it plateaus near the minimum sMSE of the full model. Approximately 20% of the total number of features are sufficient for prediction quality near the full model.

### Prediction within and across Task Variants

Predictions obtained using single task variants for training and testing are obtained in **Table 4**. When the training and test data are from the same task variant (the diagonal of the table), sMSE is approximately the same or lower than what was obtained by combining training and test data across pooled modalities. However, when training and test data are obtained from differing task variants, prediction is no better than what could be obtained by naively predicting the mean of the target distribution.

**Table 4:** Predictive ability of Gaussian Process Regression (GPR) models trained and tested on trials from single working memory task variants.

	Test task		
Training task	Auditory	Numeric	Spatial
Auditory	0.45 ± 0.06	1.15 ± 0.13	0.97 ± 0.09
Numeric	1.07 ± 0.12	0.33 ± 0.04	1.15 ± 0.13
Spatial	1.13 ± 0.14	1.11 ± 0.13	0.35 ± 0.05

*The predicted variable for all models was task load. Model performance is provided as the mean and standardized error of the mean of standardized mean square error (sMSE).*

## Discussion

We used GPR to train a model capable of accurately predicting N-back working memory load or workload. When data from all three task variants were pooled for training and testing, above chance predictions were obtained. This result is consistent with a meta-analysis of functional magnetic resonance imaging (fMRI) studies using N-back variations which showed a frontoparietal network which, although affected by the nature of the information retained, is generally active across all N-back variants (Owen et al., 2005). However, if data was trained exclusively on a single task variant, then prediction on alterative task variants was no better than a naïve model which always predicts the mean task load. Training exclusively on a single task variant may overfit to that particular variant, impairing prediction when the test variant differs. It is possible that improved cross-variant prediction could be obtained by modification of the GPR model to account for greater uncertainty in predicting a new task variant.

For the pooled data, predictive accuracy was high overall (sMSE = 0.44, *r* = 0.75), although the GPR model was less able to predict (or extrapolate to) extreme values, tending to smooth extreme values to middling values. This limitation is typical of interpolation based regressors such as GPR, especially given the limited number of data points (∼800) relative to the high dimensionality of the data (up to 192 per participant, dependant on whether any channels were removed due to excessive artifact). Extrapolation might be improved by the use of alternative covariance functions incorporating linear terms.

GPR performed significantly better than the baseline performance established by a simpler parametric technique, MLR. This was the case for models trained and tested on N-back task load, as well as for models trained and tested on subjective workload ratings provided by the participants after each block of the task. Model performance between N-back task load and subjective workload was similar, likely due to the strong relation between N-back task load and subjectively reported workload as reported in the behavioral results.

Applying feature subset selection to the model revealed that feature subsets selected based on techniques such as ANOVA are significantly less efficient at prediction than the subsets identified by GPR with ARD. For example, using the top 25% of features derived from GPR generates model performance approximately equivalent to the top 50% of features derived from an ANOVA model. Similarly, models using GPR consistently outperformed models utilizing MLR, a simpler but less flexible approach.

Periods of data containing obvious muscular artifacts were manually rejected from the dataset prior to training the machine learning model. Despite this, features in the gamma band of the EEG were most sensitive to variations in N-back level. Several works have cautioned against the use of higher frequency band power features such as beta and gamma for workload estimation (Gerjets et al., 2014; Brouwer et al., 2015), due to EMG contamination from differential motor activity in different blocks of the task. Here, the N-back task was utilized, in which mental workload between task levels is varied by task instruction rather than alteration of the perceptual or motor demands of the task. While perceptual demands do vary between the modality variants of the task utilized (auditory, numeric, spatial), as our predictor was trained and tested on a random selection of data from each of three modalities at each N-back level, the N-back level groupings do not contain systematic differences in perceptual or motor demands that would aid prediction.

As gamma band power is susceptible to contamination from muscular artifact (Muthukumaraswamy, 2013), the source of these features cannot be assumed to be of neural origin. However, the consistency of the gamma band features across participants despite the constant motor demands of the N-back suggests that, if predictions result in part due to EMG activity, more direct measures of EMG activity may prove useful for mental workload classification. It is possible that the diagnosticity of the gamma band feature is due not to a confound in how participants respond between levels of the task, but subtle postural changes on the part of participants as mental demand increases. In this sense, gamma band features may be considered an artifact when EEG is used to measure electrical activity of exclusively neural origin, but in our analysis may also represent a feature that is truly diagnostic of mental demand, and not simply an experimental design confound.

It is often desirable to minimize the number of electrode sites required for a BCI. In laboratory settings with standard electrode caps, using fewer sites can reduce experimental preparatory time or allow experimenters to allocate more time to ensuring a low-impedance connection at key sites, improving data quality. In custom-designed electrode caps, fewer sites may also reduce the size, weight, and power (SWaP) and cost of BCI systems intended to operate in real-time. Taking advantage of our non-parametric GPR model, we were able to demonstrate a method for determining subsets of channels that capture the full predictive accuracy of the entire electrode cap – and even determine the minimum number of channels, or even EEG features, required for accuracy. We observed that the 16 channels present in a commercial off-the-shelf device, mostly lateral sites near the head’s equator, capture a very large fraction of the predictive ability of the full 32 channel laboratory cap. Devices with such electrode montages might be used in future experiments, provided their EEG signal quality is acceptable.

Despite what we believe to be an overall contribution to the field, several limitations of the current report should be noted. The present paradigm used 80% of the available data for training on each cross-validation fold. This amount of training data may not be practical to acquire before a real-time device could be utilized. Additionally, our models were trained and tested within each participant. A more optimal model would be participant independent. It possible that these issues could be partially mitigated by adapting data or Gaussian Process hyperparameters that were learned from previous participants to reduce the training time required for new participants. Additionally, the present work contains data from 16 participants, all of whom are male and middle-aged. Future reports should expand workload prediction using larger and more demographically variable participant samples. Finally, while stimulus modality was randomized, participants completed increasingly demanding experimental blocks within each modality. Therefore, fatigue or tiredness could potentially contribute to estimations of mental demand.

## Conclusion

There is potentially great value in real-time, non-invasive monitoring of cognitive states by ‘passive’ BCI using methods such as electroencephalography (EEG). Cognitive variables such as workload, which are predictive of operational errors, are potentially valuable targets for real-time monitoring. Information about these variables may be useful in a variety of downstream applications, including providing situational awareness for human operators, alerting operators about high-workload situations, testing and training operators, redesign of interfaces, and redesign of working practices to optimize operator performance.

In this paper, we used EEG to monitor cognitive workload during a simple working memory task (N-back) in multiple sensory and cognitive modalities (Auditory, Numeric, and Spatial). Calibration from training data was demonstrated to be effective using GPR, out performing a more basic model utilizing MLR. GPR also provided the ability to assess the relative predictive value of each input variable (EEG electrode sites, and frequency bands at each site, together summarized as EEG ‘features’) in predicting the workload variable of interest. The GPR approach was superior to conventional analysis of variance (ANOVA) methods in determining which reduced subsets of EEG features from the training set would be most predictive about the cognitive variable of interest in the test set. This type of analysis may inform engineering efforts to produce EEG systems with few electrodes placed at the most highly informative sites on the scalp for the desired evaluations.

The current approach can be placed within a class of methods that seek to use techniques from machine learning to not only make predictions, but glean useful information about the neural or behavioral processes under study. In another example, Noh and de Sa (2014) have reported that a machine learning model trained on a subset of EEG data can be used to select features for traditional hypothesis testing on an independent test set. As this method derives candidate features for discriminating between conditions from the independent training set, it avoids the issue of multiple comparisons encountered when performing traditional hypothesis testing on several potential features within a single set of data.

In addition, in contrast to more traditional statistical methods such as MLR, the GPR approach provides confidence intervals around each prediction. Information regarding the confidence of a predictor may be useful in operational domains in order to determine when to trust the outputs of the predictive model. For example, a test point that contains data that is far outside what was observed within the training set would be predicted with a large confidence interval.

## Author Contributions

MC: Co-led research effort, collected and analyzed data, wrote software code, wrote paper. DR: Collected majority of data, wrote software code, analyzed data, contributed to writing of paper. JC: Participated in data collection, edited paper. HG: Participated in data collection, wrote stimulus/task software. MW: Initiated project plan, co-led research effort, participated in data collection.

## Conflict of Interest Statement

The authors declare that the research was conducted in the absence of any commercial or financial relationships that could be construed as a potential conflict of interest.
